# Fall predictors in older cancer patients: a multicenter prospective study

**DOI:** 10.1186/1471-2318-14-135

**Published:** 2014-12-15

**Authors:** Nathalie Vande Walle, Cindy Kenis, Pieter Heeren, Katrien Van Puyvelde, Lore Decoster, Ingo Beyer, Godelieve Conings, Johan Flamaing, Jean-Pierre Lobelle, Hans Wildiers, Koen Milisen

**Affiliations:** Department of Geriatric Medicine, Universitair Ziekenhuis Brussel, Vrije Universiteit Brussel (VUB), Brussels, Belgium; Department of General Medical Oncology, University Hospitals Leuven, Leuven, Belgium; Department of Geriatric Medicine, University Hospitals Leuven, Leuven, Belgium; Department of Public Health and Primary Care, Health Services and Nursing Research, KU Leuven, Kapucijnenvoer 35/4, 3000 Leuven, Belgium; Frailty in Ageing (FRIA) Research Group, Vrije Universiteit Brussel, Brussels, Belgium; Department of Medical Oncology, Oncologisch Centrum, Universitair Ziekenhuis Brussel, Vrije Universiteit Brussel (VUB), Brussels, Belgium; Department of Clinical and Experimental Medicine, KU Leuven, Leuven, Belgium; Beernem, Belgium; Department of Oncology, KU Leuven, Leuven, Belgium

**Keywords:** Older persons, Cancer, Falls, Geriatric assessment

## Abstract

**Background:**

In the older population falls are a common problem and a major cause of morbidity, mortality and functional decline. The etiology is often multifactorial making the identification of fall predictors essential for preventive measures. Despite this knowledge, data on falls within the older cancer population are limited. The objective of this study was to evaluate the occurrence of falls within 2 to 3 months after cancer treatment decision and to identify predictors of falls (≥1 fall) during follow-up.

**Methods:**

Older patients (70 years or more) with a cancer treatment decision were included. At baseline, all patients underwent geriatric screening (G8 and Flemish Triage Risk Screening Tool), followed by a geriatric assessment including living situation, activities of daily living (ADL), instrumental activities of daily living (IADL), fall history in the past 12 months, fatigue, cognition, depression, nutrition, comorbidities and polypharmacy. Questionnaires were used to collect follow-up (2–3 months) data. Univariate and multivariate analyses were performed to identify predictors for falls (≥1 fall) during follow-up.

**Results:**

At baseline, 295 (31.5%) of 937 included patients reported at least one fall in the past 12 months with 88 patients (29.5%) sustaining a major injury. During follow-up (2–3 months), 142 (17.6%) patients fell, of whom 51.4% fell recurrently and 17.6% reported a major injury. Baseline fall history in the past 12 months (OR = 3.926), fatigue (OR = 0.380), ADL dependency (OR = 0.492), geriatric risk profile by G8 (OR = 0.471) and living alone (OR = 1.631) were independent predictors of falls (≥1 fall) within 2–3 months after cancer treatment decision.

**Conclusion:**

Falls are a serious problem among older cancer patients. Geriatric screening and assessment data can identify patients at risk for a fall. A patient with risk factors associated with falls should undergo further evaluation and intervention to prevent potentially injurious fall incidents.

## Background

Falls are a common problem in the older population. One third of individuals 65 years and older fall at least once a year and up to half of these patients have recurrent falls [1, 2]. Fortunately, most falls do not result in any serious harm. Five to 10% of falls lead to a fracture, head injury or serious soft tissue injury [3, 4]. Additionally, accidents are the fifth leading cause of death in older persons and falls account for two thirds of these deaths due to unintentional injuries [3]. Further, the fear of a (new) fall often leads to disability. In brief, falls represent a major cause of morbidity, mortality and functional decline as well as dependency including premature nursing home admissions [1, 3].

The etiology of a fall is often multifactorial due to a combination of physiological age-related changes, pathological elements and behavioral and environmental factors [5, 6]. Therefore, identification of strong fall predictors is essential in the planning of preventive measures. Several epidemiologic studies in very heterogeneous, older populations have identified fall history as a main risk factor. Use of sedative medications, gait problems, depression, and cognitive impairment are other frequently reported determinants of falls [6, 7]. However, the effectiveness of fall screening tools based on these results remains unclear [8]. For that reason, further research in subpopulations is warranted. The data on falls in older cancer patients are limited, stating merely the presence or absence of falls rather than risk factors [9]. Seventeen of 91 newly diagnosed older cancer patients experienced at least one fall in the past 6 months following diagnosis in a study by Puts et al. [10]. Stone et al. reported that 50% of 185 adults with advanced cancer fell during a 6-month follow-up [11]. Hitcho et al. (2004) described that 74% of first falls among oncology inpatients resulted in an injury [12]. These preliminary results point to a high incidence of falls and a higher complication rate in older cancer patients compared to the data in the general population of older adults. However, they must be interpreted with caution because of the small sample sizes and high dropout rates.

The objective of this study was to evaluate the occurrence of falls among older cancer patients within 2 to 3 months after cancer treatment decision and to identify predictors for falls (≥1 fall) during follow-up.

## Methods

The methodology used in the current study has been described in detail elsewhere [13]. A brief summary is given below.

### Study design and population

This prospective, observational cohort study was performed in two academic hospitals in Belgium from October 2009 till July 2011. Patients aged 70 years and older, in whom a cancer treatment decision had to be made due to new diagnosis or disease progression/relapse, were approached by a trained nurse during a hospital visit. Depending on the time point of assessment - new diagnosis or disease progression/relapse – the treatment decision is a first decision or a subsequent decision and includes all different kinds of cancer treatment. Six tumor types were included: breast, colorectal, ovarian, lung, prostate cancer and hematological malignancies. The study was approved by the Ethical Committee of both participating hospitals (University Hospitals Leuven and University Hospital Brussels) and written informed consent (IC) was obtained by all patients or their caregiver.

### Baseline geriatric screening and assessment

The presence of a geriatric risk profile was assessed in all patients by a trained nurse using the G8 (≤14 is abnormal) and by the Flemish version of the Triage Risk Screening Tool (fTRST) (≥1 is abnormal) [13–16].

At baseline, a geriatric assessment (GA) was performed in all patients. This GA was based on the Geriatric Minimum Data Set of Clinical Trials [17] and comprised demographic and social data such as age, gender and living situation. Functional status was evaluated using Activities of Daily Living (ADL) (>6 is abnormal) [18] and Instrumental Activities of Daily Living (IADL) (<5(male)/8(female) is abnormal) [19], as well as fall history by asking the number of falls and fall-related injuries in the past 12 months, respectively.

A fall was defined as ‘an unexpected event in which the older person comes to rest on the ground, floor or lower level’ [20, 21]. Injuries were divided in two groups: minor and major. Minor injuries were defined as scrapes and scratches, bruises, superficial wounds that needed no or minimal medical attention. Major injuries were defined as sprains, severe soft-tissue bruises, severe head injuries, joint distortions and dislocations, contusions, lacerations, loss of consciousness, and fractures [22].

Cognition was evaluated using the Mini Mental State Examination (MMSE) (<24 is abnormal) [23] and risk for depression by the 15-item Geriatric Depression Scale (GDS-15) (≥5 is abnormal) [24]. Nutritional status was assessed using the Mini Nutritional Assessment-Short Form (MNA-SF) (≤11 is abnormal) [25, 26]. The Charlson Comorbidity Index (CCI) (≥1 is abnormal) [27] was used to describe the comorbidities. Self-perceived fatigue was assessed using the Mobility-Tiredness Test (Mob-T) [28] and pain was evaluated with the Visual Analogue Scale (VAS) (≥1 is abnormal) [29]. Classical oncological parameters such as Eastern Cooperative Oncology Group - Performance Status (ECOG-PS) [30], tumor characteristics (type and stage) and treatment details were recorded. The number of drugs (http://www.bcfi.be) taken during the week before inclusion was recorded to detect polypharmacy (≥5 different drugs).

### Follow-up

Occurrence of falls and fall-related injuries was registered during two to three months after cancer treatment decision. A questionnaire was sent to the patient at home, asking to self-report the variables of interest. Patients were contacted by telephone or in person in the hospital in case the questionnaire was not returned, in order to complete the follow-up data.

### Statistical analysis

Data analyses were performed using SAS v.9.3. Descriptive statistics were performed to characterize the total study population.

For comparative analyses, different groups were defined: non-fallers (no falls), fallers (≥1 fall), single fallers (=1 fall) and recurrent fallers (≥2 falls). Comparisons between groups for continuous data were done with Students’ t test, Wilcoxon test, analysis of variance or use of general linear model, as appropriate. Once significance was achieved between 2 groups, further analysis was conducted with the Tukey method. Categorical data were compared and tested with chi-square test and/or Fisher’s exact test with or without the Monte Carlo method, as appropriate. Following potential markers of falling during follow-up were analyzed at univariate level: age, gender, ECOG-PS, fTRST, G8, ADL, IADL, living situation, fall history, CCI, MOB-T for fatigue, polypharmacy, VAS for pain, MMSE, GDS and MNA-SF.

Multivariate logistic regression was performed on the patients with fully completed baseline variables. To perform this multivariate logistic regression, a multiple variable logistic model was used. Variables achieving a significance level of 0.05 in univariate analyses were entered in the multivariate analysis with stepwise variable selection and p-values to enter and to stay in the model of 0.05. Multiple collinearity was investigated with variance inflation factor (VIF).

## Results

### Patient characteristics

**Figure 1:**
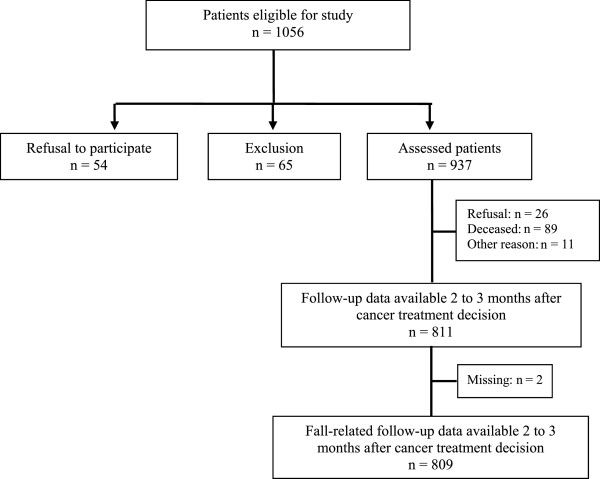
**Flow-chart of patient selection.**

To participate in the study 1056 patients were approached. Written informed consent was given by 931 patients and six caregivers, resulting in 937 patients included. At time point of follow-up, 2 to 3 months after inclusion (average of 2.38 months), fall-related data of 809 patients were available (see Figure 1).

Patient and clinical characteristics are listed in Table 1. The majority was female (595; 63.5%) and the median age was 76 years old (range 70–95). The most common malignancy was breast cancer (40.4%), followed by colorectal cancer (20.6%) and hematological malignancies (15.9%). Most participants (61.0%) had a new cancer diagnosis at inclusion and nearly half (46.1%) of patients had no comorbidities. Congestive heart failure (18.4%) and diabetes mellitus without complications (13.4%) were the most common comorbidities.

**Table 1:** Patient and clinical characteristics*

Age, years (median (range) (n = 937)		76 (70 – 95)			
			n	%	95% CI
Gender (n = 937)	- Female		595	63.5	60.4-66.6
	- Male		342	36.5	33.4-39.6
Tumor type (n = 937)	- Carcinoma	- Breast	379	40.4	37.3-43.6
		- Colorectal	193	20.6	18.0-23.2
		- Lung	73	7.8	6.1-9.5
		- Ovarian	59	6.3	4.7-7.9
		- Prostate	84	9.0	7.1-10.8
	- Hematological malignancies		149	15.9	13.6-18.2
Timepoint of assessment (n = 937)	- New diagnosis		572	61.0	57.9-64.2
	- Disease progression/relapse		365	39.0	35.8-42.1
Carcinoma (n = 788)	- Stage	- I-III	380	48.2	44.7-51.7
		- IV	408	51.8	48.2-55.3
Hematological malignancies (n = 149)	- Setting	- Curative	48	32.2	24.7-39.7
		- Palliative	101	67.8	60.3-75.3
Comorbidities	CCI (0–37) (n = 937)	- No comorbidities: score 0	432	46.1	42.9-49.3
		- Comorbidity score 1	232	24.8	22.0-27.5
		- Comorbidity score ≥ 2	273	29.1	26.2-32.0
Pain	VAS (0–10) (n = 936)	- No pain: score 0	467	49.9	46.7-53.1
		- Mild pain: score 1 – 3	144	15.4	13.1-17.7
		- Pain to treat: score ≥ 4	325	34.7	31.7-37.8
Performance status	ECOG-PS (0–5) (n = 936)	- Score 0-1	677	72.3	69.5-75.2
		- Score 2-4	259	27.7	27.3-33.1
Polypharmacy (n = 910)		- 0-4 different drugs	427	46.9	43.7-50.2
		- ≥5 different drugs	483	53.1	49.8-56.3

**Legend**: CCI: Charlson Comorbidity Index; VAS: Visual Analogue Scale; ECOG-PS: Eastern Cooperative Oncology Group – Performance Status.

*Table adapted from Kenis et al., 2014 [13].

Based on geriatric screening with G8 and fTRST, 697 (74.4%) and 773 (82.5%) patients had an abnormal screening result, respectively. Further results of the GA are described in Table 2. Abnormal nutritional status (63.7%) and presence of fatigue (60.6%) were the most frequent deficiencies. Both ADL (51.4%) and IADL (57.4%) instruments found functional dependence for half of the included patients. Nearly one third was living alone (30.2%).

**Table 2:** **Results of the geriatric screening and GA** ^**a**^

**Screening**	**Cut-off**	**Score**	**n**	**%**	**95%CI**
fTRST (0–6)	≥1	- Absence of a geriatric risk profile : score 0	164	17.5	15.1-19.9
		- Presence of a geriatric risk profile : ≤ 1	773	82.5	80.1-84.9
G8 (0–17)	≤14	- Absence of a geriatric risk profile : score > 14	240	25.6	22.8-28.4
		- Presence of a geriatric risk profile : score ≤ 14	697	74.4	71.6-77.2
**GA**	**Item/Instrument**	**Score**	**n**	**%**	**95%CI**
Living situation (n = 937)		- Living alone	283	30.2	27.3-33.1
		- Not living alone	654	69.8	66.9-72.7
Functional status	ADL (6–24) (n = 937)	- Independent: score 6	455	48.6	45.4-51.8
		- Dependent: score ≥ 7	482	51.4	48.2-54.6
	IADL (0-5/8) (n = 937)	- Independent: score 8 (female) or 5 (male)	399	42.6	39.4-45.7
		- Dependent: score < 8 (female) or 5 (male)	538	57.4	54.2-60.6
Fall history in the past 12 months	Falls^b^ (n = 937)	- No falls	642	68.5	65.5-71.5
		- 1 fall without injury	41	4.4	3.1-5.7
		- 1 fall with injury	114	12.2	10.1-14.3
		- ≥2 falls without injury	35	3.7	2.5-4.9
		- ≥2 falls with injury	105	11.2	9.2-13.2
Fatigue	MOB-T (0–6) (n = 937)	- No fatigue	369	39.4	36.3-42.5
		- Presence of fatigue	568	60.6	57.5-63.7
Cognition^c^	MMSE (0–30) (n = 936)	- Normal cognition: score ≥ 24	837	89.4	87.5-91.4
		- Mild cognitive decline: score 18–23	84	9.0	7.1-10.8
		- Severe cognitive decline: score ≤ 17	15	1.6	0.8-2.4
Depression	GDS-15 (0–15) (n = 933)	- Not at risk for depression: score 0–4	741	79.4	76.8-82.0
		- At risk for depression: score 5–15	192	20.6	18.0-23.2
Nutrition^d^	MNA-SF (0–14) (n = 937)	- Normal nutritional status: score ≥ 12	340	36.3	33.2-39.4
		- Risk of malnutrition: score 8–11	422	45.0	41.9-48.2
		- Malnourished: score ≤ 7	175	18.7	16.2-21.2

**Legend**: fTRST: Flemish version of the Triage Risk Screening Tool; GA: Geriatric Assessment; ADL: Activities of Daily Living; IADL: Instrumental Activities of Daily Living; MOB-T: Mobility – Tiredness Test; MMSE: Mini Mental State Examination; GDS: Geriatric Depression Scale; MNA-SF: Mini Nutritional Assessment-Short Form.

^a^Table adapted from Kenis et al., 2014 [13]; ^b^Injuries include minor and major injuries; ^c^Cognition: dichotomized in logistic regression in normal cognition (score 24–30) and mild/severe cognitive decline (score 0 – 23); ^d^Nutrition: dichotomized in logistic regression in normal nutritional status (score 12–14) and risk of malnutrition/malnourished (score 0–11).

### Occurrence of falls and fall related injuries at baseline and at follow-up

At baseline, 295 (31.5%) patients reported at least one fall in the past 12 months of which 219 patients (74.2%) sustained an injury. A major injury was reported by 88 patients (29.8% of fallers or 40.2% of fallers with injury).

**Figure 2:**
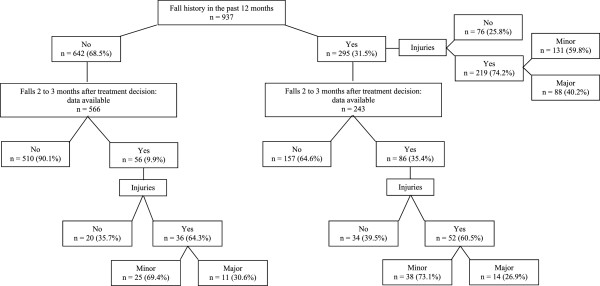
**Occurrence of falls at baseline and follow-up.**

At follow-up, a fall was reported by 142 patients (17.6%), of whom 51.4% fell more than once. Injurious falls and those resulting in major injuries were reported in 88 (62.0%) and 25 patients (17.6% of fallers), respectively. One out of every four falls with an injury was major (28.4%) (Figure 2).

Fall risk during follow-up was significantly higher in patients with baseline fall history in the past 12 months compared to those without fall history (35.4% versus 9.9%; p < 0.0001). For severity of injury, no difference was found for patients with or without baseline fall history in the past 12 months (p = 0.78).

### Univariate predictors for falls (≥1 fall)

Comparison of non-fallers with fallers (≥1 fall), single fallers (=1 fall) and recurrent fallers (≥2 falls) was possible for the following variables: age, fTRST, G8, ADL, IADL, living situation, fall history in the past 12 months, CCI, MOB-T for fatigue, polypharmacy, VAS for pain, MNA-SF and ECOG-PS. Low functionality (e.g. dependent on ADL or IADL), living alone, reported fall history in the past 12 months, geriatric risk profile (e.g. higher score on fTRST or lower score on G8), nutritional problems, high ECOG-PS, fatigue, polypharmacy and mild pain/pain to treat were all significantly associated with falling, one single fall only and recurrent falls during follow-up. Cognitive decline and risk for depression were both significantly related to falling during follow-up. The latter was also significantly associated with recurrent falls (Table 3).

**Table 3:** **Univariate predictors of occurrence of falls during follow-up**

Variable		Total population (n = 809)*	Non-fallers (no falls) (n = 667)*	Fallers (≥1 fall) (n = 142)*	Single fallers (1 fall) (n = 69)*	Recurrent fallers (≥2 falls) (n = 73)*	P-value non-fallers vs fallers	P-value non-fallers vs single fallers	P-value non-fallers vs recurrent fallers
Age	70-74	322 (39.8%)	278 (41.7%)	44 (31.0%)	23 (33.3%)	21 (28.8%)	0.0511	0.0955	0.1703
	75-79	256 (31.6%)	207 (31.0%)	49 (34.5%)	20 (29.0%)	29 (39.7%)			
	≥80	231 (28.6%)	182 (27.3%)	49 (34.5%)	26 (37.7%)	23 (31.5%)			
Gender	Female	528 (65.3%)	435 (65.2%)	93 (65.5%)	45 (65.2%)	48 (65.8%)	0.95	1.0	0.93
	Male	281 (34.7%)	232 (34.8%)	49 (34.5%)	24 (34.8%)	25 (34.3%)			
G8	No geriatric risk profile (>14)	232 (28.7%)	218 (32.7%)	14 (9.9%)	6 (8.7%)	8 (11.0%)	<0.0001	<0.0001	0.0001
	Geriatric risk profile (≤14)	577 (71.3%)	449 (67.3%)	128 (90.1%)	63 (91.3%)	65 (89.0%)			
fTRST	No geriatric risk profile (0)	153 (18.9%)	147 (22.0%)	6 (4.2%)	2 (2.9%)	4 (5.5%)	<0.0001	0.0002	0.0009
	Geriatric risk profile (≥1)	656 (81.1%)	520 (78.0%)	136 (95.8%)	67 (97.1%)	69 (94.5%)			
ADL	Independent (6)	413 (51.1%)	375 (56.2%)	38 (26.8%)	16 (23.2%)	22 (30.1%)	<0.0001	<0.0001	<0.0001
	Dependent (≥7)	396 (48.9%)	292 (43.8%)	104 (73.2%)	53 (76.8%)	51 (69.9%)			
IADL	Independent (5(male)/8(female)	367 (45.4%)	325 (48.7%)	42 (29.6%)	18 (26.1%)	24 (32.9%)	<0.0001	0.0003	0.01
	Dependent (≤4(male)/≤7(female))	442 (54.6%)	342 (51.3%)	100 (70.4%)	51 (73.9%)	49 (67.1%)			
Living situation	Not living alone	571 (70.6%)	486 (72.9%)	85 (59.9%)	42 (60.9%)	43 (58.9%)	0.002	0.0352	0.01
	Living alone	238 (29.4%)	181 (27.1%)	57 (40.1%)	27 (39.1%)	30 (41.1%)			
Fall history in the past 12 months	No falls	566 (70.0%)	510 (76.5%)	56 (39.4%)	32 (46.4%)	24 (32.9%)	<0.0001	<0.0001	<0.0001
	Falls (≥1)	243 (30.0%)	157 (23.5%)	86 (60.6%)	37 (53.6%)	49 (67.1%)			
CCI	No comorbidities (0)	388 (48.0%)	326 (48.9%)	62 (43.7%)	27 (39.1%)	35 (47.9%)	0.26	0.1230	0.88
	Comorbidities (≥1)	421 (52.0%)	341 (51.1%)	80 (56.3%)	42 (60.9%)	38 (52.1%)			
MOB-T for fatigue	No fatigue	339 (41.9%)	313 (46.9%)	26 (18.3%)	12 (17.4%)	14 (19.2%)	<0.0001	<0.0001	<0.0001
	Fatigue	470 (58.1%)	354 (53.1%)	116 (81.7%)	57 (82.6%)	59 (80.8%)			
Poly-pharmacy	Absence (0–4 drugs)	386/785 (49.2%)	338/649 (52.1%)	48/136 (35.3%)	22/66 (33.3%)	26/70 (37.1%)	0.0004	0.0037	0.02
	Presence (≥5 drugs)	399/785 (50.8%)	311/649 (47.9%)	88/136 (64.7%)	44/66 (66.7%)	44/70 (62.9%)			
VAS for pain	No pain (0)	408 (50.4%)	359 (53.8%)	49 (34.5%)	23 (33.3%)	26 (35.6%)	<0.0001	0.0012	0.003
	Mild pain/pain to treat (1–10)	401 (49.6%)	308 (46.2%)	93 (65.5%)	46 (66.7%)	47 (64.4%)			
MMSE	Normal cognition (≥24)	738 (91.2%)	615 (92.2%)	123 (86.6%)	59 (85.5%)	64 (87.7%)	0.03	0.0566	0.18
	Mild/severe cognitive decline (≤23)	71 (8.8%)	52 (7.8%)	19 (13.4%)	10 (14.5%)	9 (12.3%)			
GDS	Not at risk for depression (0–4)	663/806 (82.3%)	559/665 (84.1%)	104/141 (73.8%)	52/68 (76.5%)	52 (71.2%)	0.004	0.1095	0.006
	At risk for depression (≥5)	143/806 (17.7%)	106/665 (15.9%)	37/141 (26.2%)	16/68 (23.5%)	21 (28.8%)			
MNA-SF	Normal nutritional status (≥12)	412 (50.9%)	367 (55.0%)	45 (31.7%)	21 (30.4%)	24 (32.9%)	<0.0001	<0.0001	0.0003
	Risk of malnutrition/malnourished (≤11)	397 (49.1%)	300 (45.0%)	97 (68.3%)	48 (69.6%)	49 (67.1%)			
ECOG PS	Score 0-1	619/808 (76.6%)	529/666 (79.4%)	90 (63.4%)	42 (60.9%)	48 (65.8%)	<0.0001	0.0004	0.0073
	Score 2-4	189/808 (23.4%)	137/666 (20.6%)	52 (36.6%)	27 (39.1%)	25 (34.3%)			

**Legend**: fTRST: Flemish version of Triage Risk Screening Tool; ADL : Activities of Daily Living; IADL : Instrumental Activities of Daily Living; CCI: Charlson Comorbidity Index; MOB-T : Mobility-Tiredness test; VAS: Visual Analogue Scale; MMSE: Mini Mental State Examination; GDS: Geriatric Depression Scale; MNA-SF: Mini Nutritional Assessment - Short Form. ECOG PS: Eastern Cooperative Oncology Group - Performance Status.

*In case of different denominator, the value is mentioned in the table.

### Multivariate predictors for falls (≥1 fall) during follow-up

**Figure 3:**
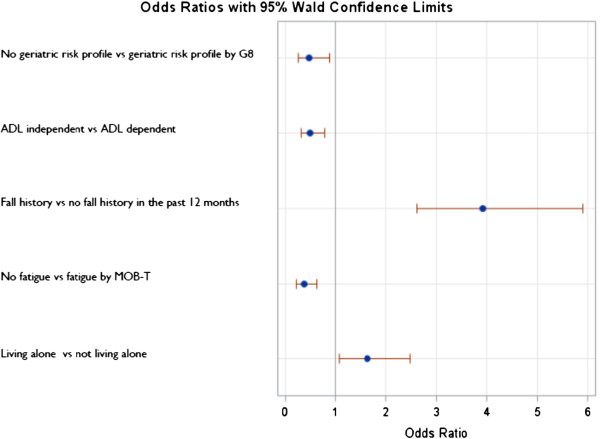
**Multivariate predictors for occurrence of falls (≥1 fall) during follow-up.**

Multivariate logistic regression was performed on the patients with fully completed baseline variables (n = 781). This regression analysis showed that falling (≥1 fall), within 2 to 3 months after cancer treatment decision can be predicted by fall history in the past 12 months, fatigue, ADL dependency, geriatric risk profile by G8 and living situation (Figure 3, Table 4). There was no collinearity between the independent variables.

**Table 4:** **Multivariate predictors of occurrence of falls (≥1 fall) during follow-up (n = 781)**

	Coefficients	Wald Chi Square	p-value		
	-1.846	130.4	<0.0001		
Selected baseline variables	Coefficients	Wald Chi-Square	p	Odds ratio’s	95% CI
Fall history in the past 12 months (fall history vs no fall history in the past 12 months)	0.684	43.3	<0.0001	3.926	2.612-5.901
ADL (ADL independent vs ADL dependent)	-0.354	9.5	0.002	0.492	0.314-0.772
MOB-T for fatigue (no fatigue vs fatigue)	-0.484	13.7	0.0002	0.380	0.228-0.634
Geriatric risk profile by G8 (no geriatric risk profile vs geriatric risk profile)	-0.377	5.5	0.019	0.471	0.251-0.883
Living situation (living alone vs not living alone)	0.245	5.2	0.023	1.631	1.071-2.482

**Legend**: ADL: Activities of Daily Living; MOB-T: Mobility Tiredness Test.

## Discussion

This study examined incidence and risk factors for falls within two to three months after a cancer treatment decision in older cancer patients. Independent fall predictors were fatigue, living alone, ADL dependency, geriatric risk profile by G8 and fall history in the past 12 months.

At baseline, one in every 3 patients reported a fall in the past 12 months, which is comparable with fall incidence in community-dwelling older adults [1, 2]. However, falls in older cancer patients might be underreported. During follow-up, 17.6% of patients had experienced already a fall, which is remarkable given the short follow-up. The current study did not find a higher fall rate for patients with advanced cancer, as reported by Stone (one in every two patients) [11]. Maybe, less frail patients with advanced cancer were included, since these were able to visit the hospital. Of note, recurrent fall rate (51.4% within follow-up) and the number of falls with major injury (29.8% of fallers at baseline; 17.6% of fallers within follow-up) were both high in comparison to international research findings among community-dwelling older adults (38.1-53.8% and 10-15%), confirming earlier findings and underlining the urgent need for effective preventive measures [1, 2, 31].

Multivariate analysis identified several independent fall predictors among older cancer patients. In accordance with other studies, fall history was the main predictor for a future fall [6, 7]. Patients with a fall history in the past 12 months at baseline were almost 4 times more likely to fall shortly after cancer treatment decision compared to patients without a fall history in the past 12 months at baseline. Another risk factor was geriatric risk profile by G8. This might be explained by the association between (history of) a fall and frailty in geriatric patients [32]. However, Puts et al. did not find a correlation between frailty markers and falls in older cancer patients [10]. Other predictors were functional capacity and fatigue, which might be inversely related. Patients with decreased functional capacity spend more effort relative to maximal ability to perform usual activities, leading to higher levels of fatigue [33]. Whereas the association between functional dependency and falls is well known [34, 35], the relation between falls and cancer/treatment-related fatigue is not well documented. However, physical fatigue may represent a risk factor for falls in older persons, since gait changes following a strenuous task are comparable to gait changes found in older persons with falls [36]. Although the effect of physical activity on cancer-related fatigue is controversial [37, 38], clinicians should promote it, since physical activity might be beneficial to maintain/improve functional capacity. The last predictor was living alone, indicating that social support in daily life after a cancer treatment decision is not only necessary for emotional health, but also for mobility issues. This is in opposition with the presumption that patients living alone are more independent and have a lower risk of falling. The identification of these fall predictors allow for targeted preventive measures based on geriatric screening and assessment data. Indeed, good prevention starts with improvement of modifiable risk factors.

Other predictors for falls in older adults, such as decreased cognition, depression, malnutrition and polypharmacy, were not identified among older cancer patients in the current study. However, there was some evidence of an effect of each that may have been too marginal to allow for inclusion into a multivariate analysis. Of course, this finding does not question the clinical relevance of these variables. As stated in the introduction, a fall is often caused by several, interacting factors. So, preventive measures in patients with high fall risk according to the identified fall predictors should be tailored considering all factors possibly contributing to higher odds of falling. According to fall risk assessment and the implementation of preventive measures, several guidelines have been developed of which the guidelines of the American Geriatrics Society ‘Prevention of falls in older persons’ [39] and the NICE guideline ‘Falls: assessment and prevention of falls in older people’ [40] are recommended.

This study has some limitations. Several risk factors for falls (e.g. vision, orthostatic hypotension, use of fall risk-increasing medication classes) were not explored. However, limiting study data to those available at the moment of treatment decision might be more relevant for clinical practice. Another limitation is the short follow-up period, possibly leading to overestimation of fall events. In addition, falls and fall history may have been underreported due to recall bias, although similarity between reported 12 month fall history at baseline and fall incidence in community-dwelling older adults supports the assumption that our data are reasonably valid and reliable. Still, future research should consider the use of a fall calendar. Furthermore, most of the included patients had a good cognition and a low number of comorbidities, suggesting that the studied sample might have been a selection of a less frail older cancer population. Strengths of this study are the prospective design, the large and heterogeneous sample, and the acceptable drop-out rate considering the population of interest.

## Conclusion

In conclusion, falls are a serious and underestimated problem among older cancer patients. Fatigue, living alone, ADL dependency, geriatric risk profile by G8 and fall history (in the past 12 months) were independent predictors of a fall. This study demonstrates that geriatric screening and assessment are useful for identifying older cancer patients at risk for falls. This allows for individually tailored interventions that might improve outcomes in this particularly vulnerable population.
